# Lower serum levels of IL-1β and IL-6 cytokines in adolescents with anorexia nervosa and their association with gut microbiota in a longitudinal study

**DOI:** 10.3389/fpsyt.2022.920665

**Published:** 2022-08-18

**Authors:** Hannah E. Specht, Nina Mannig, Meriem Belheouane, Nadia Andrea Andreani, Klaus Tenbrock, Ronald Biemann, Katrin Borucki, Brigitte Dahmen, Astrid Dempfle, John F. Baines, Beate Herpertz-Dahlmann, Jochen Seitz

**Affiliations:** ^1^Department of Child and Adolescent Psychiatry, Psychosomatics and Psychotherapy, University Hospital, RWTH Aachen University, Aachen, Germany; ^2^Max Planck Institute for Evolutionary Biology, Plön, Germany; ^3^Institute for Experimental Medicine, Kiel University, Kiel, Germany; ^4^Department of Pediatrics, Medical Faculty, RWTH Aachen University, Aachen, Germany; ^5^Department of Pediatrics, IZKF Aachen, Medical Faculty, RWTH Aachen University, Aachen, Germany; ^6^Institute for Clinical Chemistry and Pathobiochemistry, Otto-von-Guericke University Magdeburg, Magdeburg, Germany; ^7^Institute of Laboratory Medicine, Clinical Chemistry and Molecular Diagnostics, University of Leipzig, Leipzig, Germany; ^8^Institute of Medical Informatics and Statistics, Kiel University, Kiel, Germany

**Keywords:** anorexia nervosa, cytokine, proinflammatory, leaky gut, microbiome, TNF-α, IL-15, IL-6 Rα

## Abstract

**Introduction:**

Anorexia nervosa (AN) is an often chronic and debilitating psychiatric disease whose etiology is not completely understood. Recently, a potential role of inflammation has emerged in other psychiatric diseases, such as depression, PTSD and schizophrenia. The first results in adults with AN seemed to confirm a low-grade proinflammatory state until recent studies presented more differential findings. Studying adolescents with a shorter illness duration and fewer confounding factors might help elucidate the role of inflammation in the underlying pathophysiology of AN; however, the few available studies in adolescents remain ambiguous, and no longitudinal data are available in this age range.

**Methods:**

We examined the proinflammatory cytokines Tumor Necrosis Factor-alpha (TNF-α), Interleukin (IL)-1β, IL-6, IL-15, and the cytokine-receptor IL-6 Receptor alpha (IL-6 Rα) in the serum of twenty-two hospitalized female adolescent patients with AN longitudinally at admission and discharge and compared their results to nineteen healthy controls (HC). We also collected clinical data and stool samples that were analyzed with 16S rRNA amplicon sequencing to explore potential influencing factors of cytokine changes.

**Results:**

TNF-α serum levels were significantly elevated in patients with AN at admission, while IL-1β and IL-6 levels were lower at admission and discharge than in HC. After treatment, we also found significantly elevated levels of IL-6 Rα compared to HC, while IL-15 did not show significant changes. Exploratory analyses revealed positive associations of cytokine and genus-level changes between admission and discharge for IL-1β (*Bacteroides*) and IL-15 (*Romboutsia*), and negative associations for IL-15 (*Anaerostipes*) and TNF-α (uncultured Lachnospiraceae).

**Conclusion:**

We confirmed a previous finding of elevated levels of TNF-α also in adolescents with AN; however, the reduced IL-1β and IL-6 levels differed from the mostly increased levels found in adults. A mixed pro- and anti-inflammatory state appears to be present in adolescents, potentially due to their shorter illness duration. The gut microbiota, with its regulatory function on cytokine production, might play a role in mediating these inflammatory processes in AN and could offer targets for new therapeutic approaches.

## Introduction

Anorexia nervosa (AN), a serious and chronic disease of mostly female adolescents and adults, remains a disease whose etiopathogenesis is not completely clear. Diagnosis defining symptoms according to DSM-V include insufficient food intake, a fear of weight gain and a disturbed image of one’s own body (1). Genetics, neurobiology (2–4) cognition (5, 6) psychosocial and societal factors (7) have been discussed as etiological components. New research also implicates altered inflammatory pathways in patients with AN (8–10).

Inflammation in psychiatric diseases is currently being studied in several fields with increasing intensity. In adult patients with posttraumatic stress disorder and depression, elevated levels of proinflammatory cytokines, such as interleukins (IL-1β, IL-6) and tumor necrosis factor (TNF-α), were measured and linked to disease-defining neuronal patterns (11–13). In children with obsessive compulsive disorder, significantly elevated levels of TNF-α and IL-12 were reported (14), while in adolescents with depression, the role of inflammation is being debated controversially (15–17).

Similarly, research in recent years has showed evidence indicating a low-grade proinflammatory state in (mostly adult) patients with AN (9, 18). Compared to healthy controls (HC), increased IL-1β (19, 20), IL-6 (20–23) TNF-α (19, 20, 23) and IL-15 (22, 24) were found in patients’ sera, the first three also confirmed by metanalyses by Solmi et al. and Dalton et al. (9, 10). However, more recently, contradicting results in adults seem to question the narrative of uniform proinflammatory state in patients with AN: neither Caroleo et al. (25) nor Nilsson et al. (26), the latter being the largest study to date with 113 patients with AN and 114 HC, confirmed higher TNF-α, IL-1β or IL-6. Keeler et al. in the most recent, very well-controlled study of 56 patients with AN and 51 HC, even found significantly lower levels of TNF-α and IL-6 in patients (27). Thus, the amount and even direction of proinflammatory cytokine changes in adult AN appear unclear. Potential reasons for these diverging results include variations due to differing durations of illness, long-standing treatment and chronification effects, among others.

A narrative review by Gibson and Mehler summarized the signs and influencing factors of a dysregulated immune system in patients with AN with an elevated CD4/CD8 ratio, more T-cell proliferation and the previously mentioned altered inflammatory cytokine profile, differing from primary malnutrition (18). Inflammation is also modulated by the gut microbiome *via* several mechanisms, including influences during the development of the intestinal immune system in early stages of life and oral tolerance (28, 29), continuous stimulation of resident macrophages promoting regulatory T-cell induction and preventing excessive T helper 17 cell activity (30). Symbiotic intestinal microbiota appear necessary for the development and function of specific lymphocyte subsets (31). The gut microbiota also sheds immunogenic endotoxins such as lipopolysaccharides, which can, in turn, cause inflammation when traversing the gut wall (32). An altered gut microbiome has been found in adult and adolescent patients with AN and has been shown not to normalize with weight rehabilitation (33, 34), thus, likely representing more than just an epiphenomenon. The latter causal notion is supported by transplantation studies, with stool from patients with AN evoking AN-like symptoms such as weight reduction and decreased appetite in the offspring of transplanted germ-free mice compared with transplanted stool of HC (35). However, the association of inflammatory cytokines with gut microbiome changes has not been previously studied in patients with AN.

One way to gain further insight into the role of proinflammatory cytokines in the pathophysiology of AN is to study younger patients. The usually shorter illness duration and consecutively smaller rate of chronification as well as fewer attempts of treatment in adolescents potentially allow an earlier analysis of the origins and underlying processes of the disease. However, there exist only three prior studies on cytokines in adolescents with AN. They, again, showed contradicting findings of increased IL-1β and TNF-α (36) and of increased TNF-α, IL-1β and IL-6 (20) vs. lower TNF-α and unchanged IL-1β and IL-6 in acutely ill patients (37).

Furthermore, longitudinal follow-up can help gather information about the individual course of the illness and differentiate between a starvation-related state vs. illness-related trait effects. Again, previous results differ; while Nilsson et al. found no more significant differences between 114 weight-restored adult patients with AN and HC (26), Keeler et al. did report several cytokines to be different after weight restoration, including elevated TNF-α (27). In adolescent AN, no previous study has measured cytokines in a longitudinal fashion thus far.

We set out to fill this gap in the literature by longitudinally studying proinflammatory cytokines previously found to be altered in patients with AN and a cytokine receptor in a cohort of adolescent AN patients in comparison to age-matched HC. Furthermore, we explored associations of cytokine alterations with eating disorder pathology, weight-related parameters and certain taxa of the microbiome, our group found to differ between patients with AN and HC in this sample (34). We hypothesized that by studying adolescents, we might obtain a clearer picture of the direction, extent, and potentially underlying influencing factors of cytokine alterations in AN patients compared to HC early in the disease. In particular, the longitudinal analysis of adolescents with AN should help answer the question of whether cytokine alterations early in the disease are still a reversible state effect associated with starvation.

## Materials and methods

### Study sample

Our patient cohort consisted of 22 female adolescents who were treated as inpatients at the University Hospital Aachen as previously described in Schulz et al. (34). Briefly, the study’s participants were female, aged between 12 and 18 years (mean 15.94 years) with a mean BMI at admission of 15.82 (kg/m^2^). The diagnosis of AN was made according to DSM-V, and illness duration ranged between 3.17 and 63.19 months with a mean of 19.01 months. The patients were in treatment for a mean of 4.57 months. Two patients presented with an atypical AN, one with a binge/purge subtype. At admission one patient took SSRI, two patients took Olanzapin and one patient took both, at discharge four patients were medicated with SSRI, one with Olanzapin and three with both. Three patients had been treated with antibiotics within the four weeks before the stool sample was taken, so they were excluded from the association analysis comparing cytokines with the gut microbiota. One patient only provided a stool sample at admission and was therefore excluded from the explorative analyses, based on the Delta at admission to discharge. We recruited 19 age- and sex-matched HC who did not and had never suffered from any mental disease. A history of severe gastrointestinal or metabolic diseases influencing the gut microbiome were also criteria for excluding patients with AN as well as HC. A summary of the clinical data is provided in Table 1.

**TABLE 1:** Participants.

Clinical data	AN at admission (*n* = 22)	AN at discharge (*n* = 22)	HC (*n* = 19)
Age (years)	15.94 (1.87) [12.01;18.63]	16.32 (1.91) [12.24;18.99]	16.35 (1.01) [14.31;17.89]
BMI (kg/m^2^)	15.82 (2.0) [13.04;18.88]	16.31 (1.91) [16.84;20.11]	20.55 (2.26) [17.31;25.32]
BMI-SDS (z-score)	−3.08 (1.98) [−6.78;−0.59]	−0.99 (0.41) [−2.16;−0.28]	−0.41 (0.74) [−1.78;0.94]
Pre-morbid weight BMI-SDS (z-score)	−0.33 (1.06) [−3.83;1.22]		
Illness duration (months)	19.01 (14.95) [3.17;63.19]		
EDI-2 total score	293.32 (51.52) [168;372]	268.55 (66.31) [146;415]	187.89 (33.86) [123;236]
BDI-2 total score	22.50 (11.29) [0;41]	17.27 (16.21) [0;54]	5.95 (4.95) [0;17]

Values are depicted as the mean, standard deviation in brackets and minimum and maximum values in square brackets. AN, Anorexia Nervosa; BDI, Beck Depression Inventory; BMI-SDS, Body mass index—standard deviation scores; EDI, Eating Disorder Inventory; HC, Healthy Controls.

### Sample and data collection

We collected clinical data, age-adjusted standardized body mass index (BMI-SDS) based on KIGGS data calculated with Ped(z) calculator (38, 39), as well as self-report questionnaires, including the Eating Disorder Inventory 2 (EDI-2) (40) and the Beck Depression Inventory-II (BDI-II, German version) (41), at admission and discharge for patients and at one time point for HC.

Furthermore, we collected fasting blood samples of the patients and HC between seven and ten o’clock in the morning at admission and at discharge/one timepoint. Immediately afterwards, samples were centrifugated, and the supernatant was frozen at −80°C until further use to limit potential proteolysis. The concentrations of TNF-α, IL-1β, IL-6, IL-15, and IL-6 Rα were measured at the Institute for Clinical Chemistry and Pathobiochemistry, Otto-von-Guericke University Magdeburg (Magdeburg, Germany) using high-sensitive quantitative sandwich immunoassays from R&D Systems (Minneapolis, Minnesota, United States). The manufacturer’s reported sensitivity for human TNF-α (Catalog Number HSTA00E) was a mean minimum detectable dose (MDD) of 0.022 pg/ml, a MDD of 0.033 pg/ml for IL-1β (Catalog Number HSLB00D), a MDD of 0.031 pg/ml for IL-6 (Catalog Number HS600C), a MDD of 2 pg/ml for IL-15 (Catalog Number D1500) and a MDD of 6.5 pg/ml for IL-6 Rα (Catalog Number DR600). Stool samples were collected using a “stool catcher” and frozen at −80°C until 16S rRNA analysis with an updated method compared to our previous analysis in Schulz et al. (34).

Briefly, total DNA was extracted from stool samples using the DNeasy Power Soil Kit (Qiagen) following the manufacturer’s instructions. The V1-V2 region of the 16S rRNA gene were amplified using primers 27F and 338R primers using a dual barcoding. During demultiplexing, no mismatch in the barcode was allowed (Casava, Illumina). QIIME2 (v2019.10) was used to process and analyze the sequence data (42). Paired end sequences were denoised with “dada2” (43), using default parameters, unless stated: reads were truncated at the first base where the quality score dropped below *Q* = 3, the maximum number of mismatches in the overlap region was 2, the minimum length of reads after truncation was 250 bp. The obtained Amplicon Sequence Variants (ASVs) were clustered using “vsearch” with an identity of 0.97 (44). Sequences were rarefied at 17,500 reads per sample. Bacterial ASVs were annotated using the q2-feature-classifier plugin (45), using the Silva database (release 138SSU) (46). Comparison of core taxon abundances (defined as present in at least 25% of the individuals, with at least 1% relative abundance) was performed using the Mann–Whitney U and Wilcoxon signed rank tests in R.

### Statistical analysis

Statistics were calculated using SPSS Statistics, version 25.0 (IBM Corp., Armonk, NY, United States). First, we compared cytokine and receptor levels between the patients with AN at admission and at discharge and the HC, as well as the patients with AN longitudinally. Due to skewed distributions, all cytokine and receptor data were log transformed, similar to Dalton et al. (21, 22). For IL-1β values that were below the detection limit, a value of 0.0001 was used before log transformation. We used unpaired *t*-tests for approximately normally distributed cross-sectional data (at admission: IL-6, IL-6 Rα, TNF-α; at discharge: TNF-α) and Mann–Whitney-U-tests for cytokines that continued to present a non-normal distribution (at admission: IL-1β, IL-15; at discharge: IL-1β, IL-6, IL-6 Rα, IL-15, see Supplementary Table 1 for tests of normality). For the longitudinal observations between admission and discharge in AN, we calculated a paired sample *t*-test for TNF-α and Wilcoxon signed rank tests for all other variables as appropriate. All tests were carried out with a two-sided level of significance set at *p* < 0.05. To correct for multiple testing, we tested whether the results remained significant with a false discovery rate corrected *p*-value of *q* < 0.1. To rule out possible medication influences, we repeated the above analyses while excluding patients taking anti-depressant or anti-psychotic medication. Furthermore, we incorporated medication use as covariates in ANCOVA/non-parametric Quade ANCOVA to again check for independence and to quantify potential medication influences.

To test the intercorrelation of cytokine measures, we used Spearman rank correlations at admission, discharge and Delta discharge-admission.

To explore influencing factors, we correlated the cytokine and receptor levels with BMI-SDS, EDI-2 and BDI scores at admission and discharge using Spearman’s non-parametric correlation coefficient. To monitor longitudinal changes, we also assessed correlations of the differences of the mentioned variables at discharge minus admission.

Finally, we explored cytokine associations with 14 potentially altered gut microbiota genera found to differ significantly between patients with AN and HC or longitudinally between admission and discharge in AN in the above analysis, without limiting to those surviving multiple comparison correction (i.e.,. nominally significant associations were included). These were *Agathobacter, Alistipes, Bacteroides, Blautia, Christensenellaceae.R.7 group, Escherichia Shigella, Dialister, Ruminococcus.1, Subdoligranulum*, uncultured Lachnospiraceae, *Faecalibacterium, Romboutsia, Anaerostipes*, and *Fusicatenibacter* (see Table 2). To limit the amount of comparisons and due to the high interindividual differences in gut microbiota, we chose to take advantage of our longitudinal design comparing differences in cytokine/receptor levels over time with taxa abundance differences between admission and discharge only. Spearman’s correlation analyses were used in all association analyses.

**TABLE 2:** Altered abundance of microbial taxa.

Core genera	Healthy control	AN at admission	AN at discharge	AN admission versus HC *P*-value	AN discharge versus HC *P*-value	AN admission versus discharge *P*-value
Uncultured Lachnospiraceae	276.00	325.89	428.67	0.199	**0.028**	0.102
*Eubacterium coprostanoligenes* group	245.21	299.89	301.94	0.919	0.240	0.438
*Agathobacter*	186.26	223.84	392.72	0.930	0.094	**0.044**
*Alistipes*	638.84	631.32	402.89	0.872	0.062	**0.048**
*Anaerostipes*	321.32	557.05	392.94	**0.012**	0.274	**0.049**
*Bacteroides*	5346.74	6934.32	4793.06	0.204	0.466	**0.003**
*Barnesiella*	174.21	128.37	146.83	1.000	0.914	0.379
*Blautia*	398.53	435.42	562.83	0.405	**0.036**	0.170
*Christensenellaceae R7* group	311.95	408.21	96.28	0.715	**0.046**	**0.013**
Dialister	166.11	151.05	826.89	0.160	0.831	**0.015**
*Erysipelotrichaceae UCG 003*	379.26	346.74	371.83	0.704	0.682	0.619
*Escherichia-Shigella*	2115.32	99.05	836.83	**0.014**	**0.038**	0.728
*Faecalibacterium*	743.95	599.68	958.94	0.300	0.447	**0.010**
*Fusicatenibacter*	179.68	167.89	283.28	0.350	0.092	**0.025**
*Parabacteroides*	448.95	506.68	353.22	0.589	0.094	0.078
*Romboutsia*	439.53	113.68	145.06	**0.004**	**0.014**	0.571
*Ruminococcaceae UCG 002*	142.63	299.68	350.72	0.148	0.103	0.687
*Ruminococcus 1*	181.21	165.42	442.22	0.704	0.207	**0.003**
*Ruminococcus 2*	314.21	279.58	255.56	0.324	0.489	0.859
*Subdoligranulum*	361.89	205.42	623.67	0.274	0.230	**0.005**
*Sutterella*	88.16	133.63	197.83	0.481	0.628	0.650

Mean relative abundances of core genera, that showed significant differences (*p* < 0.05 uncorrected in bold) when comparing AN at admission, AN at discharge and HC. Results of Mann–Whitney-U, Wilcoxon signed rank tests.

### Ethics

This study was approved by the ethics committee of the University Hospital Aachen and carried out in accordance with the Declaration of Helsinki. All patients, parents and legal guardians gave their informed written consent for participation in our study.

## Results

### Cytokine and cytokine-receptor levels

**FIGURE 1:**
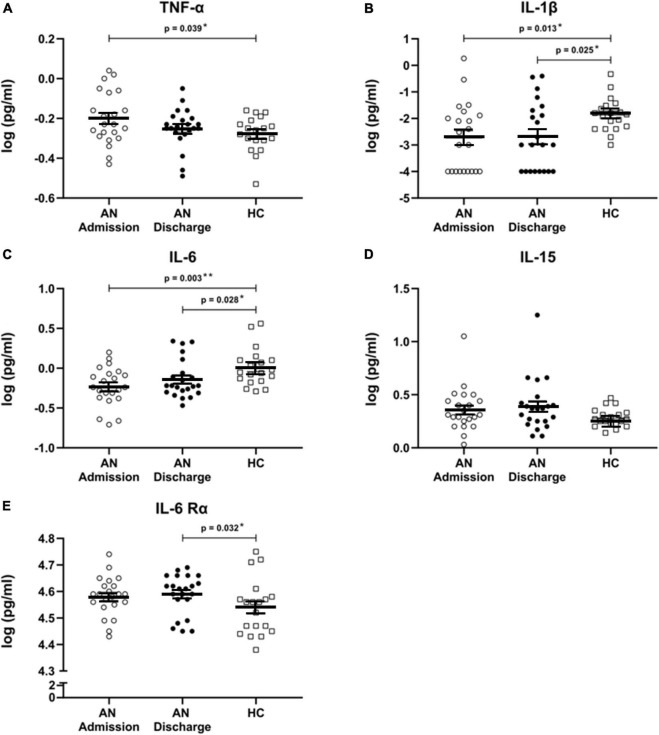
**(A–E)** Cytokines and cytokine receptor. Scatterplots showing serum cytokine levels with mean and standard error in patients with anorexia nervosa (AN) at admission to hospital, at discharge and in healthy controls. All cytokine data were log transformed. Independent *t*-tests were used to compare TNF-α at admission and discharge with HC and for IL-6 and IL-6 Rα at admission versus HC. For all other cytokines, Mann–Whitney U tests were used due to a non-normal data distribution. A paired sample *t*-test was used to compare AN at admission and at discharge for TNF-α. Wilcoxon signed rank tests were calculated for all other cytokines. All comparisons remained significant when using a false discovery rate of *q* < 0.1 to correct for multiple testing.

We found significant differences in cytokine levels between our patients at admission and the HC in IL-1β (*p* = 0.013), IL-6 (*p* = 0.003), and TNF-α (*p* = 0.039), all of which remained significant after FDR correction (Figures 1A–C). As expected, the levels of TNF-α were higher in AN patients at admission; on the other hand IL-1β and IL-6 cytokine concentrations were significantly lower in patients than in HC.

At discharge, formerly elevated TNF-α levels had approached those of HC and were no longer significantly different, while IL-1β (*p* = 0.025) and IL-6 (*p* = 0.028) remained significantly decreased. Furthermore, IL-6 Rα was significantly increased at discharge (*p* = 0.032) (Figure 1E). All analyses at discharge survived FDR correction. IL-15 levels were not significantly altered (Figure 1D).

No significant longitudinal changes were detected.

As psychotropic medicine might influence cytokine levels, we repeated above analysis excluding all patients taking psychotropic medicine. All results at admission remained significant, due to reduced power at discharge results for IL-1β remained significant, all other results did not reach statistical significance anymore (Supplementary Tables 2–4). Incorporating SSRI and antipsychotic medication as binominal covariates in an ANCOVA/non-parametric Quade ANCOVA further supported our analyses: TNF-α, IL-6 and IL-1β still differed significantly after controlling for SSRI and antipsychotic medication use at admission, while IL-6 Rα still differed significantly at discharge. All others did not differ significantly anymore, potentially for power reasons. Neither SSRI nor antipsychotic medication showed a significant influence on cytokines in these analyses (Supplementary Table 5).

### Intercorrelation of cytokines and associations with BMI-SDS, depression and eating disorder psychopathology

**FIGURE 2:**
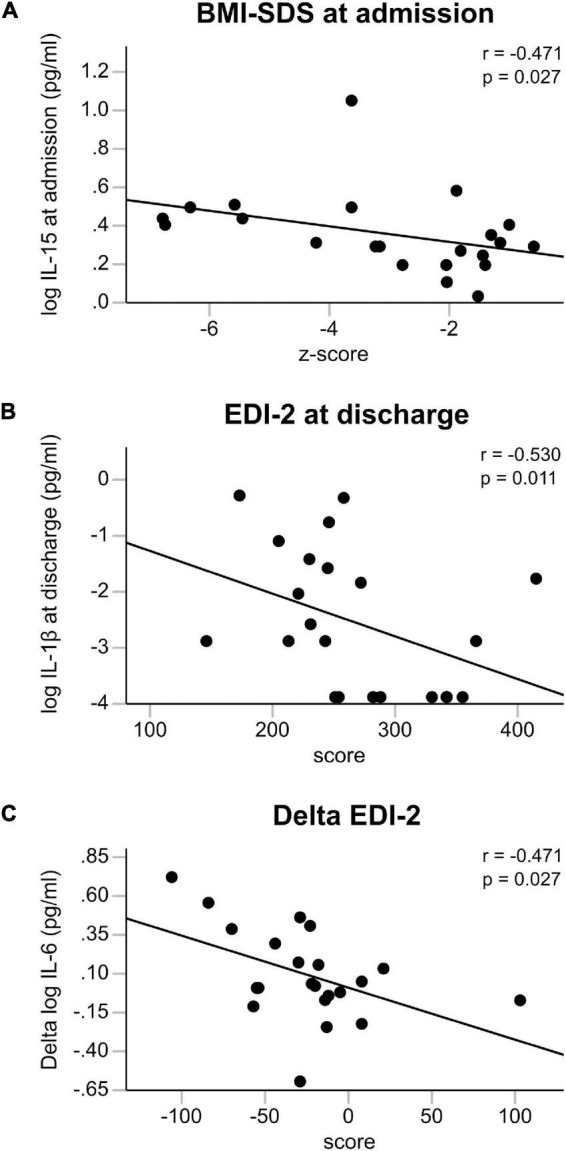
**(A–C)** Clinical associations. Association of BMI-SDS with IL-15 at admission, of IL-1β with EDI-2 at discharge and of the change in eating disorder psychopathology (EDI-2) with the change in IL-6 between admission and discharge all calculated with Spearman’s general correlation coefficient. All cytokine levels were log transformed.

There was no consistent pattern of intercorrelation between the cytokine-levels at different timepoints: IL-1β was correlated with IL-6 and IL-6 Rα at admission and with IL-6 Rα at discharge, while IL-15 correlated with TNF-α at discharge (see Supplementary Table 6). Exploring correlations of cytokine and receptor values with BMI-SDS, EDI-2, and BDI showed negative associations of IL-15 with BMI-SDS at admission (rho = −0.471, *p* = 0.027) and of IL-1β with EDI-2 at discharge (rho = −0.53, *p* = 0.011) (Figures 2A,B). Additionally, the difference between discharge and admission of IL-6 showed a negative correlation with the difference in EDI-2 (rho = −0.471, *p* = 0.027), so an increase in IL-6 was associated with a decrease in self-reported symptoms of the eating disorder psychopathology (Figure 2C).

### Associations with gut microbiota

As an exploratory approach, we examined associations of cytokines and receptors with fourteen gut bacterial genera found to potentially differ between patients with AN and the HC or longitudinally. For both parameters, we only used the differences in the values between admission and discharge in patients with AN to reduce interindividual variance and multiple comparisons.

**FIGURE 3:**
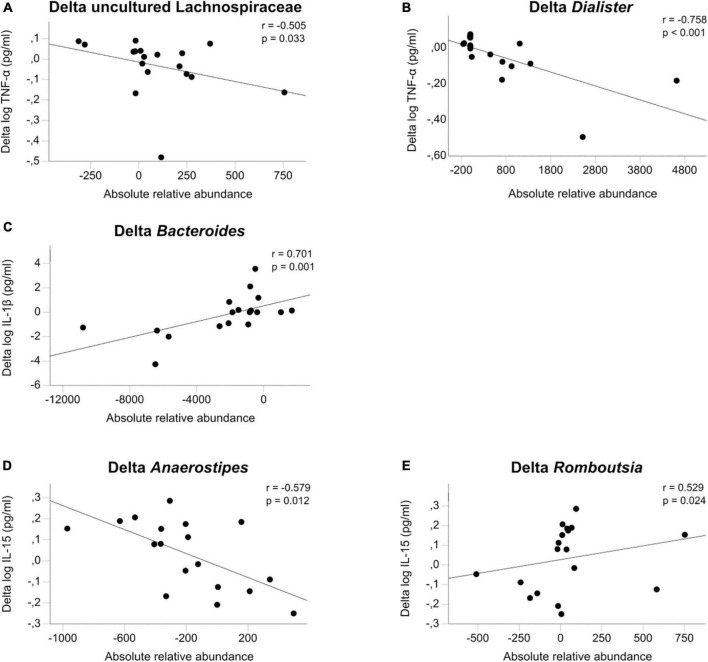
**(A–E)** Microbiome associations. Associations of the change between admission and discharge of log transformed cytokines TNF-α, IL-1β, and IL-15 with the change in gut microbiota calculated with Spearman’s general correlation coefficient. The total normalized number of reads was 17,500 reads/sample.

We found eleven significant associations in 70 comparisons, which is greater than expected by chance: TNF-α was associated with uncultured Lachnospiraceae (rho = −0.505, *p* = 0.033), *Bacteroides* (rho = 0.531, *p* = 0.023) and *Dialister* (rho = −0.758, *p* < 0.001). IL-1β was associated with *Bacteroides* (rho = 0.701, *p* = 0.001) and *Christensenellaceae R7 group* (rho = −0.51. *p* = 0.031). IL-6 was associated with *Agathobacter* (rho = 0.487, *p* = 0.04), IL-15 with *Romboutsia* (rho = 0.529, p = 0.024), *Anaerostipes* (rho = −0.579, *p* = 0.012) and *Fusicatenibacter* (rho = 0.485, *p* = 0.041). Finally, IL-6 Rα was associated with *Ruminococcus 1* (rho = 0.532, *p* = 0.023) and *Christensenellaceae R7 group* (rho = −0.52, *p* = 0.027) (see Figure 3 and Supplementary Table 7).

## Discussion

To study inflammatory changes in adolescent patients with AN early on in the disease, we analyzed four different proinflammatory cytokines and one receptor for the first time longitudinally before and after short-term weight recovery. We found significantly higher levels of TNF-α in acutely ill patients, while IL-1β and IL-6 were lower in acutely ill patients and remained reduced, even after weight recovery. IL-6 Rα was higher than HC after weight recovery, and all analyses remained significant after multiple comparison correction. Exploratory analysis revealed cytokine changes to be associated with body weight at admission and with eating disorder psychopathology and alterations in microbial taxa during weight recovery. While increased TNF-α during starvation seems to support the previously postulated low-grade proinflammatory state in acutely ill patients with AN, reduced levels of IL-1β and IL-6 do not fit this picture. Apparently, early on in the disease, there is no uniform proinflammatory reaction in adolescent patients with AN at admission, rendering pathophysiological understanding of underlying immunological processes in early AN more complex. Additionally, short-term weight recovery did not normalize these inflammatory alterations, proving them to be more than purely starvation state-related epiphenomena. Microbiome differences observed in adolescents with AN compared to adults and their changes over time might play a role in influencing these cytokine alterations.

### Cytokines

Our results confirmed most previous research in terms of elevated proinflammatory TNF-α in adults with AN also in adolescents. This effect had been found in two large metanalyses (9, 10) and several studies since (19, 20, 23, 47). However, there is also a very well-controlled recent report challenging this finding (27), and adolescent findings are mixed (20, 36, 37). Elevated TNF-α serum levels decreased during short-term weight recovery in our sample and were no longer significantly altered at discharge. Interestingly Keeler et al. also found a lower TNF-α in recovered compared to acutely ill patients, albeit on a lower absolute level (21, 27).

The role of TNF-α has always been described as versatile because of its many pathways and biological interactions (48). It is mostly known to be proinflammatory in certain diseases but also helps to (down)regulate the inflammatory process in other instances, such as infectious diseases (49, 50). TNF-α pathways of special interest with regard to AN are the effects on lipid metabolism, where it “suppresses free fatty acid uptake” and “induces lipolysis” (51). Increased TNF-α was also termed “cachectin” when first discovered, as it is the “central mediator of wasting in tumor-induced cachexia” (52). Increased TNF-α could, thus, be a weight-loss supporting and potentially mostly proinflammatory anorexia-maintaining factor, especially during acute starvation. Interestingly, in a case study, anti-TNF-α treatment, originally for a patient’s Crohn’s disease, also ameliorated comorbid AN in this patient (53). However, future research including rodent models is needed to further examine the causality of increased TNF-α for the development and maintenance of AN and could investigate potential roles in therapy, as postulated by the group of Himmerich et al. (54). Contrary to TNF-α and most previous adult studies, we found the cytokines IL-6 and IL-1β to be significantly reduced in our sample of adolescents with AN in comparison with HC and even remained so after short-term weight recovery. IL-1β and IL-6 were significantly increased in previous metanalyses (9, 10) and some recent publications (19, 47, 55); however, several current studies could not reproduce these findings or only reproduced them partially (10, 25, 47, 56). Again, findings in adolescents were mixed (20, 36, 37). Interestingly, while the study with the relatively older adolescent sample found an increase in IL-1β and IL-6 (20), in the two younger samples of the three with the shorter duration of illness, only IL-1β was increased (36) or IL-1β did not differ and IL-6 even showed a tendency to be decreased (*p* = 0.152) (37). This might point toward younger and not so chronically ill patients with AN not (yet?) showing the uniformly proinflammatory state previously found in adults.

Furthermore, changes in IL-6 during short-term weight restoration and IL-1β were inversely associated with ED psychopathological alterations in the AN group. Thus, also within the patients with AN, a higher psychopathological load was associated with even lower levels of IL-6 and IL-1β, intrinsically consistent with our finding of reduced levels of IL-6 and IL-1β in adolescent AN compared to HC.

IL-1β is a proinflammatory cytokine produced in blood monocytes after stimulation with antigens like lipopolysaccharides (LPS) and induction by other cytokines such as IL-6 and TNF-α (57). In the gut IL-1β is thus associated with intestinal inflammation and infections caused by bacteria, viruses and protozoa (58). IL-1β was found to be significantly increased in the latest meta-analysis (10) in restrictive type AN only, and not at all in other recent publications on adult AN (22, 25, 56), so results in adults are heterogeneous and its role during the course of AN remains unclear.

IL-6 is an acute phase protein, known to regulate and influence several immunological pathways, following e.g., infections or tissue damage (59). It is commonly used as early inflammation marker e.g., in neonatology to detect developing sepsis or necrotizing enterocolitis. Albeit the commonly known proinflammatory effects it is also known to act in anti-inflammatory and regulating ways (60). IL-6 plays an important role in intestinal inflammation as evidenced in animal studies. In a mouse model fetal IL-6 exposure led to gut alterations, which increased susceptibility to LPS-induced intestinal injury later in life (61). Prado et al. e.g., found fecal microbiota transplantation in necrotizing enterocolitis to reduce inflammatory markers, including IL-6 (62) supporting the proinflammatory effect of IL-6 and its interaction with gut microbiota.

As proinflammatory cytokines are produced not only by immune cells but also by adipose tissue (63), one hypothesis explaining the reduced levels of IL-6 and IL-1β could be the starvation-induced reduction of this tissue. Indeed, IL-6 has been found to be BMI dependent, e.g., in obese patients (64), and Keeler et al. found a reduction in other cytokines (IL-7 and IL-12) to be significantly associated with lowered body weight in patients with AN, which could have consecutively led to other altered cytokines downstream (27). Interestingly, Dolezalova et al. demonstrated that the mRNA expression of proinflammatory IL-6 in adipose tissue was significantly reduced in adult patients with AN (65). In our cohort, IL-6 and IL-1β were not significantly associated with BMI-SDS, making this explanation less likely. Nevertheless, IL-6 and IL-1β remained significantly decreased even after short-term weight restoration. This again supports the at least partial independence of these findings from BMI-related effects and further validates our findings. Reduced IL-6 and IL-1β could also be a result of anti-inflammatory compensatory activities. Diaz-Marsa et al. for example looked at pro- and anti-inflammatory activations in a mixed age group of eating disorder patients and found evidence for both, increased pro- and anti-inflammatory factors e.g., p38/mitogen-activated protein kinases (MAPK) and extracellular signal-regulated kinase (ERK)/MAPK ratios to be significantly elevated (56).

IL-6 Rα was significantly higher in patients after weight restoration than in HC (and marginally so, before). Previous studies in adults with AN with elevated IL-6 showed decreased levels of IL-6 Rα (9, 66, 67), so increased IL-6 Rα in our sample might correspond to the reduced IL-6 values we found. To our knowledge, this is the first time that IL-6 Rα has been measured in adolescents with AN.

IL-15 levels were nominally high in our study, in line with the proinflammatory increases found in adults with AN (22, 24); however, differences from HC did not reach significance in our relatively small sample. IL-15 serum levels were inversely associated with BMI-SDS at admission, indicating higher values in more affected patients. This tendency, together with increased TNF-α, could be interpreted as a sign of a coexisting partial proinflammatory state already present in adolescent patients with AN.

### Gut microbiota

As the gut microbiome has a known regulatory effect on inflammatory processes in the gut wall and the host, we explored cytokine associations with seven microbial taxa that were previously found to be altered in our sample of adolescent patients with AN compared with HC. To reduce interindividual variations and multiple testing, we focused on comparing cytokine changes following short-term weight restoration with parallel changes in microbial taxa. To our knowledge, this is the first time this connection has been analyzed in patients with AN. We showed eleven nominally significant associations, three positive (Delta *Dialister* with Delta TNF-α, Delta *Bacteroides* with Delta IL-1β and Delta *Romboutsia* with Delta IL-15) and two negative (Delta *Anaerostipes* with Delta IL-15 and Delta TNF-α with Delta uncultured Lachnospiraceae) that are depicted in Figure 3. The idea of an association between psychiatric diseases and the gut has been described in the model of the gut-brain-axis (29, 68). This axis allows bidirectional communication between several structures in the gut, such as the gut microbiota, the enteric nervous system, the mucosa, and the central nervous system (CNS), *via* afferent and efferent autonomic pathways (69). Another direct link are bacterial metabolites such as short-chain fatty acids that affect enterocytes and gut permeability but can also traverse the blood–brain barrier to affect neurons and astrocytes (70). Last, inflammation and immune processes stemming from the gut are thought to affect the brain *via* cytokines and migrating immune cells (71). The exact effects of gut microbiota on the different enteric target structures are the subject of more recent research (72, 73).

In our sample, changes in proinflammatory cytokines were inversely associated with microbial taxa with known anti-inflammatory properties, such as *Lachnospiraceae* (74, 75) and *Anaerostipes* (76, 77). The rise of the former one during short-term weight recovery could be partly responsible for the reduction seen in TNF-α. *Lachnospiraceae* and their ability to produce butyrate have been associated with positive effects on cardiovascular risk factors (78) and anti-inflammatory effects in inflammatory bowel disease (79). Furthermore, Schulz et al. found that higher *Lachnospiraceae* levels at admission predicted shorter durations of treatment, a known indicator of good outcome, even after controlling for other known predictors (34). Conversely, *Dialister* was reported to correlate to pro-inflammatory disease activity in spondyloarthritis (80) and Veillonaceae (the family *Dialister* belongs to) is known to be associated with pro-inflammatory events (81) and with increased inflammation in cirrhosis (82). Thus, bidirectional interdependence between gut microbiota and inflammation in AN appears possible.

### Potential mechanisms and implications of partially reduced proinflammatory cytokines in adolescent anorexia nervosa

The most puzzling finding of our study is the partially increased (TNF-α and trend IL-15) and at the same time partially reduced (IL-6, IL-1β) proinflammatory status in adolescents with AN. Potentially, a longer illness duration is needed to express the full picture of a more uniform proinflammatory state seen in most adult patients with AN (but see Keeler et al. for a mixed picture also in a well-controlled study in adults with AN) (27). Possible explanations for the reduced cytokines could include differences in the production and influence of regulators of cytokine production and regulatory pathways due to starvation. For example, a lack of N-caspase 1 is described to lead to a lack of activation of IL-1β (83, 84). Another aspect might be evidence that shows higher IL-1β levels after ovulation (85), as most of our patients had amenorrhea in contrast to the HC. One can also take into consideration that most patients suffering from AN avoid nutrition with a high caloric value, such as fat (86). These eating habits might influence cytokine levels as well. For certain nutrients, an anti-(87, 88) or proinflammatory (89) effect has already been proven. However, none of these aspects appear to primarily explain a difference in adolescents versus adults, as all of these effects could be expected to play a similar role in both age groups.

Chronification might still be the driving factor behind these differences, as patients in our sample presented much shorter illness durations with a mean of 19.01 months (SD ± 14.95) in comparison to adults, for example, in Dalton et al. with 11.68 years (SD ± 12.2) (21). Alternatively, age itself has been described as a factor for increasing cytokine levels (90, 91), which might play an interacting role in explaining the lower levels in adolescents as well. During development of body and brain, AN might, thus, have a differential impact on inflammation during adolescence than during adulthood, potentially explaining the differing findings although all studies were compared to age-matched healthy controls.

### Limitations

First, our sample size is small. Associations of cytokines with microbial taxa can thus only be exploratory. However, finding significant associations already in our small sample can be an interesting starting point informing future studies. Larger studies with an even longer follow-up after discharge could help to validate our findings and address remaining discrepancies with the existing literature on adolescents with AN. Second, it needs to be considered that there are other psychiatric comorbidities present in many patients with AN, such as depression or anxiety, which makes a distinction of cause and effect between the altered cytokines and the different diseases more difficult (13). Larger sample sizes could also allow correction for comorbidities. Third, findings of major depression disorder indicate an influence of antidepressant drugs on the alterations of cytokines (92). We thus repeated our comparisons between the patients with AN and the HC and found no influence of SSRI or antipsychotic administration at admission, supporting our findings. The power was too low to reproduce all findings at discharge, only the results for IL-1β remained significant.

## Conclusion

This is the first study to follow cytokines in adolescents with AN longitudinally. We found consistently reduced levels of IL-6 and IL-1β, while TNF-α was increased during acute starvation only, thus, presenting a mixed picture of pro- and anti-inflammatory signaling. A potential reason for this difference to more proinflammatory states found in adults could be shorter illness duration or developmental factors attributed to lower age. Microbiome changes could play a mediating role in the influence of the gut-brain axis on inflammation. Future research should aim to test inflammatory states in adolescents with AN in a larger sample and with an even longer follow-up period. A better understanding of the underlying inflammatory processes in AN could open up the way for new therapeutic approaches modulating inflammation directly, e.g., by anti-inflammatory medication, or indirectly *via* microbiome-targeted interventions.

## Data availability statement

The raw data can be obtained by contacting the data availability committee at KJP-Data-Access@ukaachen.de with a reasonable request.

## Ethics statement

The studies involving human participants were reviewed and approved by the Ethics Committee of the Medical Faculty RWTH University Aachen. Written informed consent to participate in this study was provided by the participants’ legal guardian/next of kin.

## Author contributions

BH-D and JS designed the concept of the study. JS, BH-D, and BD coordinated the patients’ enrollment. HS and NM recruited HC and collected the study samples and clinical data. RB and KB analyzed the serum samples. MB and JB preanalyzed the stool samples and NA did the final analysis. HS did the statistical analysis supervised by JS and AD. HS interpretated the results, assisted by JS, NA, and KT. HS wrote the initial manuscript, revised by JS and corrected by all other co-authors. All authors approved the submitted version.
