# Supplementary material for: Lower serum levels of IL-1β and IL-6 cytokines in adolescents with anorexia nervosa and their association with gut microbiota in a longitudinal study
Source: Front Psychiatry. 2022 Aug 18;13:920665. doi: 10.3389/fpsyt.2022.920665 (PMC9433656; doi:10.3389/fpsyt.2022.920665)
Supplement: Supplementary file 1 [file Data_Sheet_1.docx]

Supplementary Material

**Supplementary Table 1.**

Normality tests for the inflammatory markers according to the Shapiro-Wilk test method in AN at admission (A), AN at discharge (B) and in HC (C).

A)

| Log Inflammatory marker  AN at admission | Statistic | Df | p-value |
| --- | --- | --- | --- |
| TNF-⍺ | 0.956 | 22 | 0.413 |
| IL-1β | 0.864 | 22 | **0.006** |
| IL-6 | 0.966 | 22 | 0.618 |
| IL-15 | 0.869 | 22 | **0.007** |
| IL-6 R⍺ | 0.974 | 22 | 0.797 |

B)

| Log Inflammatory marker  AN at discharge | Statistic | Df | p-value |
| --- | --- | --- | --- |
| TNF-⍺ | 0.918 | 22 | 0.069 |
| IL-1β | 0.873 | 22 | **0.009** |
| IL-6 | 0.865 | 22 | **0.006** |
| IL-15 | 0.810 | 22 | **0.001** |
| IL-6 R⍺ | 0.892 | 22 | **0.020** |

| Log Inflammatory marker  HC | Statistic | Df | p-value |
| --- | --- | --- | --- |
| TNF-⍺ | 0.928 | 19 | 0.162 |
| IL-1β | 0.968 | 19 | 0.744 |
| IL-6 | 0.904 | 19 | 0.059 |
| IL-15 | 0.958 | 19 | 0.532 |
| IL-6 R⍺ | 0.936 | 19 | 0.222 |

C)

**Supplementary Tables 2-5.**

Group comparisons of cytokine levels of AN vs. HC at admission (S3) and discharge (S4) and AN longitudinal (S5) without SSRI (A), without antipsychotic (B), without SSRI or antipsychotic (C) medication and ANCOVAs including SSRI and antipsychotic medication (S6).

S2 Excluding patients with medication at admission
A B C

| Inflam-matory marker | AN at admission without SSRI  (n = 15) | AN at admission - HC |  | Inflam-matory marker | AN at admission without Olanzapin (n = 18) | AN at admission - HC |  | Inflam-matory marker | AN at admission without SSRI and Olanzapin (n = 14) | AN at admission - HC | |
| --- | --- | --- | --- | --- | --- | --- | --- | --- | --- | --- | --- |
| Log | Mean (SE) | p-value |  | Log | Mean (SE) | p-value |  | Log | Mean (SE) | p-value | |
| TNF-⍺ | -0.1969 (0.03101) | **0.041 *** ^a^ |  | TNF-⍺ | -0.1913 (0.03223) | **0.032 *** ^a^ |  | TNF-⍺ | -0.189 (0.03398) | **0.032 *** ^a^ | |
| IL-1β | -2.8168 (0.25009) | **0.008 *** ^b^ |  | IL-1β | -2.8546 (0.26058) | **0.008 *** ^b^ |  | IL-1β | -2.791 (0.26714) | **0.013 *** ^b^ | |
| IL-6 | -0.2326 (0.0563) | **0.004 *** ^a^ |  | IL-6 | -0.2411 (0.05748) | **0.003 *** ^a^ |  | IL-6 | -0.2375 (0.06064) | **0.005 *** ^a^ | |
| IL-15 | 0.3713 (0.04753) | 0.086 ^b^ |  | IL-15 | 0.3639 (0.05054) | 0.183 ^b^ |  | IL-15 | 0.3733 (0.05251) | 0.113 ^b^ | |
| IL-6 R⍺ | 4.58 (0.01698) | 0.173 ^a^ |  | IL-6 R⍺ | 4.5752 (0.01683) | 0.232 ^a^ |  | IL-6 R⍺ | 4.5751 (0.01779) | 0.245 ^a^ | |
| Abbreviations:  HC-healthy control; IL-interleukin; SE-standard error; TNF-tumor necrosis factor;  ^a^-p-value T-test; ^b^-p-value Mann-Whitney-U-Test; *-significant p < 0.05 | | | | | | | | | | |  |

| Inflam-matory marker | AN at discharge without SSRI  (n = 15) | AN at discharge - HC |  | Inflam-matory marker | AN at discharge without Olanzapin (n = 18) | AN at discharge - HC |  | Inflam-matory marker | AN at discharge without SSRI and Olanzapin (n = 14) | AN at discharge - HC | |
| --- | --- | --- | --- | --- | --- | --- | --- | --- | --- | --- | --- |
| Log | Mean (SE) | p-value |  | Log | Mean (SE) | p-value |  | Log | Mean (SE) | p-value | |
| TNF-⍺ | -0.2529  (0.02895) | 0.497 ^a^ |  | TNF-⍺ | -0.2568  (0.02547) | 0.545 ^a^ |  | TNF-⍺ | -0.2573  (0.03073) | 0.591 ^a^ | |
| IL-1β | -2.6762 (0.27458) | **0.026 *** ^b^ |  | IL-1β | -2.6929  (0.28363) | **0.025 *** ^b^ |  | IL-1β | -2.7275 (0.28976) | **0.022 *** ^b^ | |
| IL-6 | -0.1077 (0.06348) | 0.14 ^b^ |  | IL-6 | -0.1074  (0.06137) | 0.114 ^b^ |  | IL-6 | -0.0999 (0.06767) | 0.19 ^b^ | |
| IL-15 | 0.3787 (0.07231) | 0.348 ^b^ |  | IL-15 | 0.3823 (0.06356) | 0.3 ^b^ |  | IL-15 | 0.3756 (0.0776) | 0.476 ^b^ | |
| IL-6 R⍺ | 4.5941 (0.02012) | **0.042 *** ^b^ |  | IL-6 R⍺ | 4.5936 (0.01644) | **0.023 *** ^b^ |  | IL-6 R⍺ | 4.5881 (0.02062) | 0.058 ^b^ | |
| Abbreviations:  HC-healthy control; IL-interleukin; SE-standard error; TNF-tumor necrosis factor;  ^a^-p-value T-test; ^b^-p-value Mann-Whitney-U-Test; *-significant p < 0.05 | | | | | | | | | | |  |

S3 Excluding patients with medication at discharge
A B C

S4 Excluding patients with medication comparing admission and discharge

A B C

| Inflam-matory marker | AN at admission - AN at discharge without SSRI  (n = 15) |  | Inflam-matory marker | AN at admission - AN at discharge  without Olanzapin  (n = 18) |  | Inflam-matory marker | AN at admission - AN at discharge without SSRI and Olanzapin  (n = 14) |
| --- | --- | --- | --- | --- | --- | --- | --- |
| Log | p-value |  | Log | p-value |  | Log | p-value |
| TNF-⍺ | 0.242 ^d^ |  | TNF-⍺ | 0.09 ^d^ |  | TNF-⍺ | 0.205 ^d^ |
| IL-1β | 0.675 ^c^ |  | IL-1β | 0.95 ^c^ |  | IL-1β | 0.61 ^c^ |
| IL-6 | 0.307 ^c^ |  | IL-6 | 0.102 ^c^ |  | IL-6 | 0.198 ^c^ |
| IL-15 | 0.57 ^c^ |  | IL-15 | 0.554 ^c^ |  | IL-15 | 0.778 ^c^ |
| IL-6 R⍺ | 0.394 ^c^ |  | IL-6 R⍺ | 0.267 ^c^ |  | IL-6 R⍺ | 0.3 ^c^ |

Abbreviations:
HC-healthy control; IL-interleukin; TNF-tumor necrosis factor;
^c^-p-value Wilcoxon-Test; ^d^-p-value pair sample T-test; *-significant p < 0.05

S5 A-C Parametric ANCOVAs including SSRI and antipsychotic medications as covariates at admission. D-F Non-Parametric Quade ANCOVAs including SSRI and antipsychotic medications as covariates at discharge.

A)

| Tests of intersubject effects |  |  |  |  |  |
| --- | --- | --- | --- | --- | --- |
| Dependant Variable: Log TNF-⍺ at admission | | | | | |
| Source | Typ III square sum | df | Mean of squares | F | Sig. |
| corrected model | 0.071^a^ | 3 | 0.024 | 1.739 | 0.176 |
| constant term | 2.042 | 1 | 2.042 | 150.18 | <0.001 |
| SSRI at admission | 0.000 | 1 | 0.000 | 0.013 | 0.909 |
| Olanzapin at admission | 0.009 | 1 | 0.009 | 0.642 | 0.428 |
| Patient_Control | 0.069 | 1 | 0.069 | 5.089 | 0.030 |
| Error | 0.503 | 37 | 0.014 |  |  |
| Total | 2.850 | 41 |  |  |  |
| Corrected total variation | 0.574 | 40 |  |  |  |

B)

| Tests of intersubject effects |  |  |  |  |  |
| --- | --- | --- | --- | --- | --- |
| Dependant Variable: Log IL-1β at admission | | | | | |
| Source | Typ III square sum | df | Mean of squares | F | Sig. |
| corrected model | 10.459^a^ | 3 | 3.486 | 3.311 | 0.030 |
| constant term | 211.415 | 1 | 211.415 | 200.76 | <0.001 |
| SSRI at admission | 0.700 | 1 | 0.700 | 0.664 | 0.420 |
| Olanzapin at admission | 1.210 | 1 | 1.210 | 1.149 | 0.291 |
| Patient_Control | 9.780 | 1 | 9.780 | 9.287 | 0.004 |
| Error | 38.963 | 37 | 1.053 |  |  |
| Total | 272.024 | 41 |  |  |  |
| Corrected total variation | 49.422 | 40 |  |  |  |

C)

| Tests of intersubject effects |  |  |  |  |  |
| --- | --- | --- | --- | --- | --- |
| Dependant Variable: Log IL-6 at admission | | | | | |
| Source | Typ III square sum | df | Mean of squares | F | Sig. |
| corrected model | 0.599^a^ | 3 | 0.200 | 3.291 | 0.031 |
| constant term | 0.505 | 1 | 0.505 | 8.319 | 0.007 |
| SSRI at admission | 0.000 | 1 | 0.000 | 0.002 | 0.962 |
| Olanzapin at admission | 0.019 | 1 | 0.019 | 0.309 | 0.582 |
| Patient_Control | 0.589 | 1 | 0.589 | 9.703 | 0.004 |
| Error | 2.244 | 37 | 0.061 |  |  |
| Total | 3.417 | 41 |  |  |  |
| Corrected total variation | 2.843 | 40 |  |  |  |

D) Log IL-1β at discharge

| Quade Nonparametric Analysis of Covariance | | | |
| --- | --- | --- | --- |
| F | DFH | DFE | P Value |
| 1,789 | 1 | 30 | 0.191 |

| Pairwise Comparisons of Groups | | | |
| --- | --- | --- | --- |
| Comparison | t | DF | P Value |
| 1 vs. 2 | -1.338 | 30 | 0.191 |

E) Log IL-6 at discharge

| Quade Nonparametric Analysis of Covariance | | | |
| --- | --- | --- | --- |
| F | DFH | DFE | P Value |
| 0.544 | 1 | 33 | 0.466 |

.

| Pairwise Comparisons of Groups | | | |
| --- | --- | --- | --- |
| Comparison | t | DF | P Value |
| 1 vs. 2 | -0.738 | 33 | 0.466 |

| Quade Nonparametric Analysis of Covariance | | | |
| --- | --- | --- | --- |
| F | DFH | DFE | P Value |
| 5.372 | 1 | 33 | 0.027 |

F) Log IL-6 R⍺ at discharge

.

| Pairwise Comparisons of Groups | | | |
| --- | --- | --- | --- |
| Comparison | t | DF | P Value |
| 1 vs. 2 | 2.318 | 33 | 0.027 |

**Supplementary Table 6.**

Intercorrelation of cytokine measures with Spearman rank correlations at admission (A), discharge (B) and Delta discharge-admission (C).

A)

| Log  Inflammatory marker  AN at admission | IL-1β | IL-6 | IL-6 R⍺ | IL-15 | TNF-⍺ |  |
| --- | --- | --- | --- | --- | --- | --- |
| IL-1β |  | 0.670 | 0.614 | -0.025 | 0.408 | Correlation coefficient |
| IL-6 | **0.001** |  | 0.336 | 0.018 | 0.269 |  |
| IL-6 R⍺ | **0.002** | 0.126 |  | -0.422 | 0.281 |  |
| IL-15 | 0.911 | 0.938 | 0.051 |  | 0.064 |  |
| TNF-⍺ | 0.059 | 0.225 | 0.205 | 0.776 |  |  |
|  | p-value | | | | |  |

B)

C)

| Log  Inflammatory marker  AN Delta  discharge-admission | IL-1β | IL-6 | IL-6 R⍺ | IL-15 | TNF-⍺ |  |
| --- | --- | --- | --- | --- | --- | --- |
| IL-1β |  | 0.425 | 0.604 | -0.122 | 0.387 | Correlation coefficient |
| IL-6 | **0.048** |  | 0.303 | -0.434 | -0.072 |  |
| IL-6 R⍺ | **0.003** | 0.170 |  | -0.378 | 0.251 |  |
| IL-15 | 0.588 | **0.043** | 0.083 |  | 0.155 |  |
| TNF-⍺ | **0.075** | 0.751 | 0.259 | 0.490 |  |  |
|  |  |  | p-value |  |  |  |

| Log  Inflammatory marker  AN at discharge | IL-1β | IL-6 | IL-6 R⍺ | IL-15 | TNF-⍺ |  |
| --- | --- | --- | --- | --- | --- | --- |
| IL-1β |  | 0.176 | 0.490 | 0.250 | 0.128 | Correlation coefficient |
| IL-6 | 0.434 |  | 0.226 | -0.206 | 0.132 |  |
| IL-6 R⍺ | **0.021** | 0.311 |  | 0.111 | 0.045 |  |
| IL-15 | 0.261 | 0.358 | 0.623 |  | 0.472 |  |
| TNF-⍺ | 0.569 | 0.558 | 0.844 | **0.027** |  |  |
|  |  |  | p-value |  |  |  |

**Supplementary Table 7.**
Correlation of Delta discharge-admission of cytokines and nominally significant core genera with Spearman rank correlations.

| **Delta cytokines** | **Delta TNF-⍺** | | **Delta IL-1β** | | **Delta IL-6** | | **Delta IL-15** | | **Delta IL-6 R⍺** | |
| --- | --- | --- | --- | --- | --- | --- | --- | --- | --- | --- |
| **Correlation Spearman - Rho** | rho | p-value | rho | p-value | rho | p-value | rho | p-value | rho | p-value |
| **Delta uncultured Lachnospiraceae** | **-0.505** | **0.033** | 0.111 | 0.662 | 0.288 | 0.246 | -0.106 | 0.674 | 0.023 | 0.929 |
| **Delta *Agathobacter*** | 0.217 | 0.387 | 0.420 | 0.083 | **0.487** | **0.04** | -0.138 | 0.584 | 0.410 | 0.091 |
| **Delta *Alistipes*** | 0.214 | 0.395 | -0.215 | 0.392 | -0.265 | 0.287 | -0.121 | 0.633 | 0.059 | 0.817 |
| **Delta *Anaerostipes*** | -0.292 | 0.240 | -0.036 | 0.886 | 0.042 | 0.868 | **-0.579** | **0.012** | 0.370 | 0.130 |
| **Delta *Bacteroides*** | **0.531** | **0.023** | **0.701** | **0.001** | 0.247 | 0.324 | 0.009 | 0.971 | 0.342 | 0.165 |
| **Delta *Blautia*** | -0.055 | 0.829 | -0.052 | 0.838 | 0.044 | 0.861 | -0.09 | 0.723 | 0.205 | 0.414 |
| **Delta *Christensenellaceae R7 group*** | -0.156 | 0.537 | **-0.510** | **0.031** | -0.435 | 0.071 | 0.327 | 0.185 | **-0.520** | **0.027** |
| **Delta *Dialister*** | **-0.758** | **<.001** | -0.225 | 0.369 | 0.180 | 0.475 | -0.109 | 0.666 | -0.040 | 0.875 |
| **Delta *Escherichia-Shigella*** | 0.179 | 0.478 | -0.156 | 0.538 | -0.020 | 0.938 | 0.067 | 0.791 | -0.447 | 0.063 |
| **Delta *Faecalibacterium*** | 0.162 | 0.521 | -0.198 | 0.431 | -0.313 | 0.206 | 0.373 | 0.128 | -0.366 | 0.135 |
| **Delta *Fusicatenibacter*** | -0.161 | 0.523 | -0.360 | 0.143 | -0.089 | 0.726 | **0.485** | **0.041** | -0.163 | 0.518 |
| **Delta *Romboutsia*** | -0.121 | 0.633 | -0.166 | 0.510 | -0.044 | 0.861 | **0.529** | **0.024** | -0.018 | 0.945 |
| **Delta  *Ruminococcus 1*** | -0.004 | 0.987 | 0.023 | 0.927 | 0.081 | 0.751 | -0.256 | 0.305 | **0.532** | **0.023** |
| **Delta *Subdoligranulum*** | -0.187 | 0.458 | -0.273 | 0.273 | -0.360 | 0.142 | -0.141 | 0.576 | 0.007 | 0.977 |
